# Evaluation of a Concept Mapping Task Using Named Entity Recognition and Normalization in Unstructured Clinical Text

**DOI:** 10.1007/s41666-020-00079-z

**Published:** 2020-10-16

**Authors:** Sapna Trivedi, Roger Gildersleeve, Sandra Franco, Andrew S. Kanter, Afzal Chaudhry

**Affiliations:** 1 Cambridge Clinical Informatics, NIHR Cambridge Biomedical Research Centre, Cambridge University Hospitals NHS Foundation Trust, Hills Road, Cambridge, England UK; 2 Intelligent Medical Objects (IMO), Rosemont, IL USA

**Keywords:** Natural language processing, Named entity recognition, Clinical letters, Gold standard, Text mining, Annotation

## Abstract

In this pilot study, we explore the feasibility and accuracy of using a query in a commercial natural language processing engine in a named entity recognition and normalization task to extract a wide spectrum of clinical concepts from free text clinical letters. Editorial guidance developed by two independent clinicians was used to annotate sixty anonymized clinic letters to create the gold standard. Concepts were categorized by semantic type, and labels were applied to indicate contextual attributes such as negation. The natural language processing (NLP) engine was Linguamatics I2E version 5.3.1, equipped with an algorithm for contextualizing words and phrases and an ontology of terms from Intelligent Medical Objects to which those tokens were mapped. Performance of the engine was assessed on a training set of the documents using precision, recall, and the F1 score, with subset analysis for semantic type, accurate negation, exact versus partial conceptual matching, and discontinuous text. The engine underwent tuning, and the final performance was determined for a test set. The test set showed an F1 score of 0.81 and 0.84 using strict and relaxed criteria respectively when appropriate negation was not required and 0.75 and 0.77 when it was. F1 scores were higher when concepts were derived from continuous text only. This pilot study showed that a commercially available NLP engine delivered good overall results for identifying a wide spectrum of structured clinical concepts. Such a system holds promise for extracting concepts from free text to populate problem lists or for data mining projects.

**Electronic supplementary material:**

The online version of this article (10.1007/s41666-020-00079-z) contains supplementary material, which is available to authorized users.

## Introduction

The use of electronic health records (EHRs) has transformed patient care by facilitating easier access to organized healthcare data, making service delivery safer and more efficient [1, 2].

The retrieval and analysis of data stored within EHRs have the potential to drive further improvement in patient care. Data derived from structured fields (for example, coded data such as diagnoses, medications, allergies, and lab results) have successfully been used for reporting healthcare outcomes and generating alerts and best practice advisories to guide treatment decisions [3, 4]. However, there is a vast amount of data not captured in structured fields. These data are locked in free text records such as clinical letters, discharge summaries, and radiology reports. These clinical narratives often provide information missing from the structured fields on diagnoses and outcomes of treatment, but manual review of the free text can be labor intensive to undertake and even inaccurate.

Natural language processing (NLP) provides an automated method of analyzing free text with the potential advantage of being more time and cost efficient than manual review [5]. NLP systems have successfully been used to identify concepts from free text and transform them into structured data to establish disease status [6], identify drug-drug interactions [7], and detect adverse events. [8] NLP has also been used to improve efficiency and accuracy in identifying outcomes such as cancer recurrence and disease flares in chronic conditions [9, 10].

A rule-based NLP system deconstructs sentences and identifies grammar concepts by assigning a part of speech (e.g., noun, adjective) to each word, identifying noun phrases, and applying pronoun resolution and other linguistic rules to interpret the meaning of the sentence [11]. The words or phrases (tokens) are then mapped to terms in a dictionary of clinical terms. Together, the tokens may differ from the mapped term with respect to word order, word variants, abbreviations, acronyms, synonyms, punctuation, misspellings, and words that do not influence the meaning of the underlying concept. Linguistic analysis of the tokens combined with a lexically rich dictionary allows for normalization of heterogeneous natural language representations to standard terms. For the purposes of this study, we have used the IMO Core Terminology (a proprietary concept-orientated terminology system using clinical diagnosis and problem list vocabulary mapped to standard code sets such as ICD-10 codes, SNOMED CT, and others).

The ability to document a structured problem list in electronic records is necessary in many contexts for good longitudinal patient care, and if used as intended, it provides a means to capture pertinent information about a patient’s important diagnoses and symptoms in an accessible format. However, these lists are often not kept up-to-date [12, 13] and therefore lead to delays in service delivery and potentially compromise patient care [14]. The use of NLP to improve problem list documentation has been previously explored; however, most earlier studies involved extracting a small defined set of features (clinical entities) from a limited set of notes from a single clinical specialty [15–17], limiting their validity for generalized use across a healthcare enterprise for any patient with any condition. For example, Meystre and Haug use UMLS MetaMap Transfer (MMTx) and a negation detection algorithm called NegEx to extract medical problems for inclusion in the problem list. They considered an array of clinical documents such as pathology reports, radiology reports, progress notes, and discharge summaries, but only considered 80 diagnoses [18]. More recently, NLP has successfully been used in conjunction with machine learning models to automatically generate more complete problem lists in comparison with the EHR problem lists [19] supporting the use of NLP for the identification of generalized medical concepts.

Informing clinical decision-making with problem list entries requires that the information accurately captures the intent of the clinicians who have documented such findings or arrived at specified diagnoses. While clinicians are not perfect in this regard, expecting a computerized system to do so without adding or amplifying mistakes is unrealistic. However, when extracting concepts from archival documents, it might be useful to have a tool that presents candidate structured entries to a human reviewer alongside marked-up text. While this still requires effort by a clinician or appropriately trained abstractor, it promises greater efficiency than reading the entirety of the text and then searching through a dictionary of structured terms. Such a tool could also be implemented in a real-time application in which a clinician creates a free text narrative (via typing or dictation) and the engine analyzes the document, presenting the extracted and coded concepts as candidates for addition to the problem list. Such an approach could both preserve narrative storytelling in the record and expedite encoded data entry.

In this pilot study, we investigated the feasibility and accuracy of using a commercial NLP engine enhanced with clinical interface terminology to extract and normalize mapping the identified terms to unique disorders, findings, clinical situations, family history, and historical procedures from unstructured clinical notes.

## Methods

### Study Setting and Clinical Documents

The work was approved for completion as part of a service evaluation. The study setting was Cambridge University Hospitals (CUH) NHS Foundation Trust, an academic facility providing routine and tertiary care. New patient codes were used to identify patients attending initial outpatient clinic visits in 2014. To ensure that the narrative text included a range of clinical content and accounted for differences in length and style of writing in medical and surgical letters, sixty clinic letters were selected randomly from five different specialties (colorectal surgery, gastroenterology, geriatrics, nephrology, and neurosurgery) and anonymized. The specialties were equally represented by selecting 12 notes from each of them. The letters were transcriptions of verbal dictations that averaged 385 words each. They consisted primarily of unstructured narrative in paragraph form. Several letters also included numbered lists of items enriched in formal diagnoses or historical procedures, often employing abbreviations and including narrative commentary; for example:

1. Hypertension 1997
2. Type II diabetes 2010 no known microvascular disease
3. Biventricular failure with CRT
4. PAF 2014

### Intelligent Medical Objects Core Terminology

IMO Core Terminology, previously known as IMO Problem (IT), contains 330,000 concepts, representing states of being that are relevant to health or healthcare, including normal states. Each concept is represented by one canonical term and may be associated with multiple others so long as they are all exactly synonymous. The synonyms display a wide range of lexical variation, including regional spelling differences, misspellings, and abbreviations. For example, the IMO concept “malignant neoplasm of left breast” has a synonym “left breast CA.” The synonymous terms support normalization to standard terms. IMO terms also have human-curated mappings to most ICD-10 code systems, SNOMED CT, and the SNOMED UK extension to support analytics and billing.

### Development of Gold Standard Guidance

Gold standard guidelines for identifying IMO concepts in the text of a note had previously been developed using a separate set of notes from the same clinics by two clinicians. Annotators searched using an IMO browser for IMO terms exactly synonymous with concepts represented in the span of each sentence. Only the most specific instance of each concept was annotated. For example, in the sentence, “She noticed that her left hand developed some numbness and pain,” the token string “left hand … numbness” matched to the IMO concept “numbness of left hand” and the string “left hand … pain” to “left hand pain.” Each identified IMO concept was then assigned to one of five semantic categories (disorder, clinical finding, situation affecting health, family history, or history of procedure), one of three degrees of certainty (asserted, uncertain, or denied), and one of four temporalities (current, historical, future, or abstract). The guidance provided instructions with numerous examples on these steps and dozens of other points, including (1) allowing word variants to match text to IMO terms (e.g., “slurring his speech” is exactly synonymous with “slurred speech”), (2) allowing exact clinical synonyms to match text to IMO terms (e.g., “piles” is exactly synonymous with “hemorrhoids”), (3) not inferring the presence of a more specific IMO concept without its explicit statement in the text (e.g., “he was *alert* and eating breakfast” would not be matched to the IMO term “*mentally* alert”), (4) not allowing words with near synonymous meaning to match text to IMO terms (e.g., “good left ventricular function” would not match to “normal left ventricular function”), and (5) not annotating concepts too vague or ambiguous to be clinically useful (e.g., “disorder,” “fall,” “trauma”), even if present in the browser. The gold standard guidance was revised to its final version after the inter-annotator agreement step described below.

### Annotation Tool

The Multi-document Annotation Environment (MAE) tool [20] was used to mark up text representing IMO concepts, including discontinuous spans. Text was labeled as “discontinuous” if the words forming the embedded concept were separated by additional words without semantic significance. For example, the text “…has had diarrhoea which at times is extremely watery” represents the IMO concept of “watery diarrhea” but is labeled discontinuous as the essential components “watery” and “diarrhea” are separated by nonessential words. Each text span was recorded along with the matching IMO term, its semantic type, temporality, and certainty. There were three choices for assigning certainty: denied (words such as “no” and “without” negated the concept), uncertain (words such as “possible” and “maybe” modified the concept), and asserted (the author or a subject in the text positively asserted the concept, as in “she does have postural hypotension” or there were no qualifications as to the existence of the concept).

**Fig. 1:**
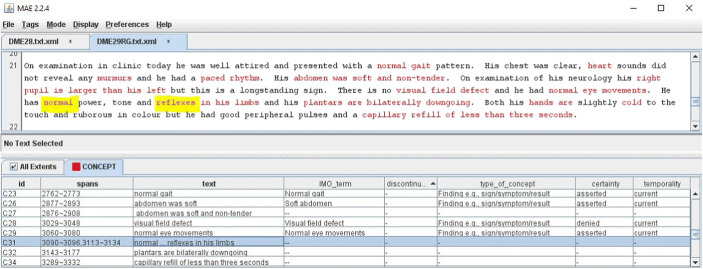
Screenshot of MAE annotation tool. An example of the unstructured text is shown with the text spans in which concepts are embedded identified in red and an example of a discontinuous concept highlighted in yellow. The table shows the text excerpt, the matching IMO term, and attributes including semantic type and assessment of negation

Every instance of a concept was annotated even if present multiple times in the text. When the annotator identified a span of text that was a common clinical utterance that is clinically useful but could not be found in the browser, the text was annotated but not matched to any IMO term (see Fig. 1). These terms were reviewed later for addition to IMO content. For example, the phrase “non-visible hematuria,” commonly used in the UK but not in the USA, was added as a synonym on the IMO concept “microscopic hematuria.”

### Linguamatics I2E Software

We employed Linguamatics’ Interactive Information Extraction Platform (I2E), to develop a query that matches text features to IMO’s Core Terminology. The I2E platform allowed us to create a specific query, defined by the criteria we set, to identify relevant concepts (see Online Resource 1 for an EASL representation of the query). The I2E engine then retrieved the information from the text using indexing techniques, and based on the output, it was possible to adjust the query within I2E to improve the performance.

The following steps were undertaken:

1. Loading the IMO Core Terminology system into I2E.
2. Indexing the clinical notes against the IMO terminology. I2E assigned part-of-speech tags to each word and chunked the words into noun and verb phrases. These chunks were then mapped to terms from the IMO dictionary.
3. Selecting indexing options built into I2E that affect how the chunks are mapped to IMO dictionary terms. The options we chose a priori allowed the engine to:

i. Expand conjunctions (match terms even when separated by a conjunction)
ii. Perform fuzzy matching (navigate alphanumeric combinations, hyphenation, brackets, and slashes)
iii. Uppercase error correction (including uppercase letters when correcting misspellings)
iv. Not restrict location in the text (match IMO terms in any syntactic arrangement)
v. No shorter matches which makes sure that the longest strings possible are matched
vi. Allow for morphological variance

(4) Creating a query to assign the following labels to the identified IMO terms: negated, uncertain, historical, and family history.
(5) Creating a blacklist of IMO concepts that would not be displayed in the results even if present in the text and appropriately mapped by the engine. This aligned with gold standard guidance that deemed some concepts too general to be of utility for populating problem lists (e.g., disorder, mass, swelling) or lent themselves to false positive results (e.g., ADD, cold, cavity).

### Study Design

**Fig. 2:**
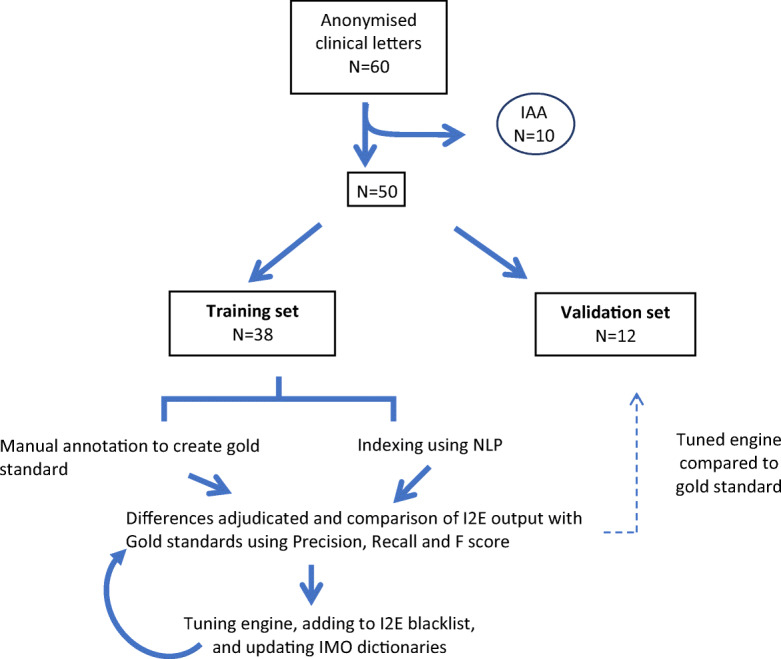
Study design showing allocation of notes for IAA, training set, and validation set and process for evaluation of training set

Sixty clinic letters were anonymized from which 10 letters were randomly selected and used exclusively to determine the inter-annotator agreement. Thirty-eight of the remaining 50 documents were annotated separately by the two clinicians as a training set and run through the I2E engine. The specialties were roughly evenly represented across the 38 documents. Precision, recall, and the F1 score were used as performance measures. The remaining 12 documents were then used to validate the results. The inter-annotator agreement (IAA) set, training set, and test set all capture the same range of semantic types and have comparable distribution of semantic types, with the exception of there being more semantic type “disorder” items and less semantic type “finding” items in the test set as compared with the IAA set or the training set (Fig. 2).

### Inter-annotator Agreement for Gold Standard

Ten of the 60 notes were set aside to determine the reliability of the gold standard guidelines. The IAA was calculated using precision, recall, and the F1 measure, with one annotator arbitrarily assigned as the equivalent of the gold standard [21, 22] This was done with a strict expectation of exact synonymy between annotators. The analysis was repeated with a relaxed expectation in which agreement was deemed to occur when both annotators identified the same core concept, but one included additional specificity mentioned in the text that the other missed. For example, if one annotator recorded “diarrhea” but the other had identified the concept “watery diarrhea” in the text, a false negative result was scored under the strict expectation but a true positive under the relaxed one. Disagreements between the two clinicians were adjudicated with assistance from a third party trained in linguistics but not clinical medicine.

### Training Set

The 38 training documents were run through I2E, and the results were aligned with the gold standard in accordance with their locations in the text. Each clinician analyzed the results for the other’s documents, assigning true positive (TP), false positive (FP), and false negative (FN) scores for each row, using both strict and relaxed criteria. A single I2E result was scored both as FP and FN if it misrepresented the embedded concept; for example, the result “alcoholic intoxication” derived from the text “does not drink any alcohol” was an FP for obvious reasons but also an FN because it missed the gold standard’s IMO term “does not drink alcohol.” If the I2E result captured only part of the concept (i.e., was “broader than”), it was scored as false negative under strict requirements and true positive under relaxed ones; for example, if the engine output was “bleeding,” but the gold standard concept was “blood on toilet paper,” the result was scored a strict FN but a relaxed TP.

For each result scored as a TP in which the IMO term and the I2E result were exact string matches, the result was allowed to stand without further review. All other results were reviewed by the two clinicians and the non-clinician to confirm TP, FP, and FN scoring. Gold standards are occasionally inaccurate, and changes may be required to them based on further review. When all agreed that the gold standard contained an error, the standard was revised, scoring changed, and editorial refined as appropriate. When all agreed that the original assigned score was incorrect for whatever reason, the score was corrected. Precision, recall, and the F1 measure were calculated for results, both with strict and relaxed standards, with a subgroup analysis by semantic type.

#### Engine Tuning

The second round of analysis of the training set was aimed at “engine tuning.” All 38 documents were reprocessed in a second round in which the morphological variance indexing option was turned off. It was deemed impractical to repeat the analysis for the other 6 indexing options (refer to the “Linguamatics I2E Software” section above). Based on ad hoc exploration of the options, combined with preliminary understanding of the nature of the FPs, this single change was thought to be most likely to improve precision. Another dimension of “engine tuning,” also aimed at improving precision, consisted of adding IMO terms to the blacklist function in I2E.

#### Content Tuning

During review of both rounds of analysis, it became apparent that “synonyms” on IMO concepts in rare cases were not exactly synonymous and led to FPs (e.g., the term “strain” had been listed as a synonym for “muscle strain” and resulted in an FP). Adding or manipulating IMO terms was termed “content tuning.”

A final round of analysis of the training set was performed with the modified indexing options, the updated blacklist, and IMO content changes.

### Test Set

A similar analysis was performed on 12 test documents using the optimal indexing options along with an updated blacklist and with IMO content tuning. After the strict and relaxed scores were determined at the conceptual level, the results were then analyzed with respect to the negation flag. Thus, the final metrics were precision, recall, and the F score using both strict and relaxed synonymy standards, with additional analysis for negation and discontinuous text for each semantic type. The negation analysis consisted of comparing the annotator’s annotation as to whether the concept was denied, uncertain, or asserted and the engine’s assignment of corresponding flags. For example, if the annotator marked the concept as uncertain or denied and the engine identified the concept but flagged it as asserted, it was scored as a false positive. If the annotator deemed that that the concept was asserted but the engine flagged is an uncertain or denied, it was scored as a false negative.

## Results

### Inter-annotator Agreement for Annotating 10 Set-Aside Documents

When annotating 10 set-aside documents, the F1 score for inter-annotator agreement with a strict synonymy requirement for all semantic types was 0.79. For subtypes, the F1 score was 0.95 for disorders; 0.79 for finding; 0.77 for situations, not calculable for family history; and 0.67 for historical procedures (see Table 1).

**Table 1:** Inter-annotator agreement. Precision, recall, and F1 score for concepts of different semantic types, with a strict expectation of matching synonymy and a relaxed expectation

	Precision	Recall	F1 score
Strict agreement			
Overall (all semantic types)	0.82	0.77	0.79
Disorder	0.94	0.96	0.95
Finding	0.95	0.68	0.79
Situation affecting health	0.92	0.67	0.77
Family history	0.00	N/A^a^	N/A^a^
History of procedure	1.00	0.50	0.67
Relaxed agreement			
Overall	0.88	0.78	0.83
Disorder	1.00	0.96	0.98
Finding	1.00	0.70	0.82
Situation affecting health	1.00	0.68	0.81
Family history	1.00	1.00	1.00
History of procedure	1.00	0.50	0.67

^a^The recall and F score for family history were not calculable because there were no true positives with the strict requirement

### Gold Standard for Training and Test Sets

The original gold standard for the training phase consisted of 672 concepts. In the first round of analysis on the development set, the engine identified an additional 19 concepts in the text that were not identified by the annotators (2.75%). For example, the engine matched the text “renovascular disease” to the IMO term “vascular disorder of kidney,” but the annotator did not. These were added to the gold standard, for a total of 691 concepts, of which 473 only occurred once in the corpus. The final gold standard for the test phase consisted of 212 concepts, 158 of which only occurred once in the corpus.

### Training Phase Results

The I2E multiple query was run on the training set of 38 documents and yielded 721 results for scoring. The number of scored results is greater than the number of concepts in the gold standard because of the presence of false positives.

Table 2 shows the results of the overall scores in the training data across all semantic types (disorders, findings, situation affecting health, family history, and history of procedure).

**Table 2:** Training data. True positives (TP), false positives (FP), false negatives (FN), precision, recall, and F1 scores when assessing for accuracy of negation, with strict and relaxed matching expectations

	Matching standard	Scored items = 721					
		TP	FP	FN	Precision	Recall	F1
Overall without negation	Strict	506	20	199	0.95	0.72	0.82
	Relaxed	545	17	166	0.97	0.77	0.86
Overall with negation	Strict	470	52	207	0.90	0.69	0.78
	Relaxed	508	45	175	0.92	0.74	0.82

### Test Phase Results

The overall results with and without assessing accuracy of negation from the 12 documents used in the testing phase consisting of 243 scored items are shown in Table 3. Table 4 shows the data using continuous text only. Table 5 shows the data broken down by semantic type, including negation, continuous, and discontinuous data with Table 6 showing the results for continuous data only.

**Table 3:** Test data. True positives (TP), false positives (FP), false negatives (FN), precision, recall, and F1 scores when assessing for accuracy of negation, with strict and relaxed matching expectations

	Matching standard	Scored items = 243					
		TP	FP	FN	Precision	Recall	F1
Overall without negation	Strict	165	17	61	0.91	0.73	0.81
	Relaxed	174	17	52	0.92	0.77	0.84
Overall with negation	Strict	145	36	62	0.81	0.70	0.75
	Relaxed	152	38	53	0.80	0.75	0.77

**Table 4:** Test data using continuous data only. True positives (TP), false positives (FP), false negatives (FN), precision, recall, and F1 scores when assessing for accuracy of negation, with strict and relaxed matching expectations

	Matching standard	Scored items = 221					
		TP	FP	FN	Precision	Recall	F1
Continuous text without negation	Strict	162	17	42	0.91	0.80	0.85
	Relaxed	167	17	37	0.91	0.82	0.87
Continuous text with negation	Strict	143	35	43	0.81	0.77	0.79
	Relaxed	146	37	38	0.80	0.80	0.80

**Table 5:** Test data results broken down by semantic type with requirement of accurate negation, including spans of discontinuous text, with strict and relaxed matching expectations

	Scored items	Matching standard	TP	FP	FN	Precision	Recall	F1
Disorder	112	Strict	80	23	20	0.78	0.8	0.79
		Relaxed	87	24	12	0.78	0.88	0.83
Family history	5	Strict	5	0	0	1.00	1.00	1.00
		Relaxed	5	0	0	1.00	1.00	1.00
Finding	70	Strict	47	7	26	0.89	0.65	0.75
		Relaxed	47	8	25	0.87	0.66	0.75
History of procedure	15	Strict	5	3	11	0.63	0.31	0.42
		Relaxed	5	3	11	0.63	0.31	0.42
Situation affecting health	12	Strict	8	3	5	0.73	0.62	0.67
		Relaxed	8	3	5	0.73	0.62	0.67
Disorder + finding	182	Strict	127	30	46	0.81	0.74	0.77
		Relaxed	134	32	37	0.81	0.79	0.80

**Table 6:** Test data results broken down by semantic type with requirement of accurate negation, excluding spans of discontinuous text, with strict and relaxed matching expectations

	Scored items	Matching standard	TP	FP	FN	Precision	Recall	F1
Disorder	104	Strict	80	22	13	0.78	0.86	0.82
		Relaxed	83	23	9	0.78	0.9	0.84
Family history	5	Strict	5	0	0	1.00	1.00	1.00
		Relaxed	5	0	0	1.00	1.00	1.00
Finding	61	Strict	45	7	17	0.88	0.74	0.8
		Relaxed	45	8	16	0.87	0.75	0.8
History of procedure	14	Strict	5	3	10	0.63	0.33	0.43
		Relaxed	5	3	10	0.63	0.33	0.43
Situation affecting health	10	Strict	8	3	3	0.73	0.73	0.73
		Relaxed	8	3	3	0.73	0.73	0.73
Disorder + finding	165	Strict	125	29	30	0.82	0.81	0.81
		Relaxed	128	31	25	0.81	0.84	0.83

## Discussion

In this pilot study, we assessed the ability of a rule-based NLP system to extract clinically meaningful concepts from clinical free text using a clinical interface terminology. This was a named entity recognition task in which the Linguamatics NLP engine parsed text into chunks and mapped them to IMO’s concept-based dictionary, which contains up to dozens of synonymous terms for each concept—essentially a gazette. The potential strength of this system resides in the linguistic parsing and analysis available in the engine combined with the depth and breadth of the dictionary to which tokens are matched. The contextual feature of negation was also assessed, but analysis was not performed for accuracy of the engine’s historical flagging in this pilot project. Semantic tagging without negation flagging is still an important function since identifying narrative that discusses presence or absence of a feature may focus attention on a subset of materials for review. The system performed well compared with other evaluations in the literature, especially given the breadth and depth of the structured vocabulary source [23].

A modest expectation for performance would be for the engine to extract concepts of all semantic types, but limited to contexts in which they were represented in continuous text strings, without an expectation of accurate flagging of negation, and with a relaxed expectation of synonymous matching. For this, which might be appropriate for some indexing use cases, the F score was 0.87. A higher performance expectation involves discontinuous text spans, requires accurate negation, and demands exactly synonymous matching. With these requirements, the F score was 0.75, still good performance.

### Differences Among Semantic Types

The results differed by semantic type. Limiting the analysis to diagnoses and clinical findings, the F score was 0.81 using strict criteria, even when accurate negation was required so long as the concept was represented with continuous text. The numbers of concepts categorized as family history, situation affecting health, and historical procedures were small, so interpretation of the F1 scores in these categories should be made with caution. A lesson learned from this pilot is that the historical procedure semantic type (e.g., “history of cholecystectomy”) is particularly challenging. Representing the historical dimension of the act in the structured concept itself (i.e., using the words “history of” or “status post”) is likely not the best way to capture this data for this task. Rather, extracting procedure concepts themselves (e.g., “cholecystectomy”) from the text and flagging them as historical would better align with clinical terminologies, including IMO, which has a distinct procedure domain not used for this project. This would require significant expansion of annotation guidance, dictionary loading, and analysis.

### Precision and Recall of IMO-I2E Engine

The precision of the system was generally much better than the recall, reflecting the low numbers of false positives. The number of false negatives was higher than expected. Some false negatives were not surprising (e.g., the engine did not extract the IMO concept “performs activities of daily living (ADL) independently” from the text “independent of all ADL’s”), but others were. For example, some exact string matches between the text and IMO terms were missed (e.g., “lower abdominal pain”). In some cases, stop words, linking verbs, and pronouns were the only evident differences between the text and the IMO terms. For example, the engine did not identify the IMO term “feeling exhausted” in the sentence, “She says she feels exhausted most of the time.” The IMO term “normal neurological exam” was not extracted from the text “neurological examination was normal.” The IMO term “lives with husband” was not tagged in “lives with her husband,” and “uses wheelchair” was not found in the tokens “uses a wheelchair.” The cases in which the engine mishandled these structures were greatly outweighed by the cases in which it successfully matched similar text to concepts. As a next step beyond this pilot study, investigating this behavior would significantly improve performance and should be readily addressed by simple improvements to the indexing query.

The difficulties in interpreting medical text are well documented and include the use of synonyms, abbreviations, misspellings, nonstandard English terms, and actual errors in documentation. This is reflected in the difficulty of achieving the IAA consistently above 0.9 for all semantic types despite extensive collaboration on creating and following annotation guidance. This project was especially challenging for annotators and the engine because the clinical concepts involved were not limited to a particular specialty or problem type: the entire range of clinical medicine was in scope and the task was not limited to formal disorders and included normal findings. Expecting a machine to do better seems unrealistic. Nonetheless, the engine detected concepts that the annotators missed, indicating that automated systems can offer advantages over manual annotation.

Allowing the annotators to record concepts that were not found in the source terminology has been described before [22]. The annotators noted 42 concepts embedded in the text that were not present in the IMO dictionary. Only 2 of the concepts missing from IMO were present in SNOMED CT. Based on a subjective assessment of their generalizability and utility, 12 of these were added to the IMO corpus but did not factor into the analysis (e.g., “central adiposity,” “blunting of right costophrenic angle,” “disc-osteophyte complex”). Beyond treating this as a workflow issue, it highlights the potential for the annotation process itself to enrich the universe of structured data. While time consuming and imperfect, manual annotation is a way to fill conceptual gaps in terminology systems using real-world data.

### Limitations and Comparisons with Other Tools

The limitations of this work include the small sample size, especially for semantic types other than disorders and findings. It was performed at a single site, and the format of the notes was similar even though they spanned five very different specialties. The study was not sufficiently powered to differentiate performance across the different specialties, but as we conducted the evaluation, we did not perceive an obvious trend towards more false positive or negative results in one specialty versus another. This might be an area for future research. The annotation guidance and the query were imperfectly aligned with respect to identifying historical concepts and treatment of concepts that are rendered by the author as a hypothetical, abstract, or future entity (e.g., “Most patients with established cervical spondylotic myelopathy… will develop progressive neurological symptoms as time goes by”). The software tools used in this project (the I2E query builder, IMO terminology browser, MAE annotation tool, and Excel) were distinct applications that required a large amount of manually recording, moving, and analyzing data. An end-to-end system that combines the needed functionality into a single workspace for the user would greatly enhance the efficiency of the process. This was designed as a proof of concept study, and therefore, further work with a larger corpus of notes would be required to further validate the NLP engine.

As noted in the “Introduction,” Meystre and Haug used UMLS MMTx and the NegEx negation detection algorithm to extract medical problems for addition to the problem list [18]. Their study, employing a wider variety of clinical note types but with a more limited scope of 80 diagnoses, achieved a recall of 0.74 and a precision of 0.76 with a default data set.

Perhaps the most similar study to our own in is a pilot study conducted by Devarakonda et al. in which they sought to automatically generate a problem list from EHR data [24], but unlike Meystre and Haug, they did not limit the concepts extracted to a subset of diseases. They used IBM Watson to extract all clinical concepts belonging to the semantic groups “Disorders,” “Procedures,” “Physiology,” and “Living Beings.” However, the key difference from our study being that they chose to optimize recall based on “the assumption that it is easier for physicians to reject non-problems presented to them than to search for true problems buried in the vast amount of data” . Increasing recall at the expense of precision resulted in mixed feedback from clinicians. They appreciated the ability of the system to find problems that may have been otherwise overlooked, but were keenly aware of the problem of “increased noise level” [24].

The tool MetaMap, which was built to map biomedical text to the UMLS, was also used by St-Maurice and Kuo to extract de-identified primary care concepts and map them to UMLS codes to understand emergency room use. The 417 concepts that were extracted were categorized as “biological symptoms,” “diagnosis,” “psychological,” “social,” “drugs,” “regional oddities,” “EMR oddities,” or “other.” No precision and recall were reported. Instead, the authors only extracted concepts that met certain statistical criteria, thus excluding many more concepts [25].

In addition to MetaMap, frequently used tools to extract clinical concepts from text are cTAKES and MedLEE. MedLEE is mainly used for pharmacovigilance and pharmacoepidemiology, but cTAKES has been implemented in a variety of use cases, such as the identification of patient cohorts, extraction of adverse drug events, and detection of medication discrepancies [26]. However, it is said that cTAKES “achieves high recall (at the cost of low precision) by identifying all phrases that have any potential to be a relevant concept” [27]. The tool CliNER, which uses a word- and character-level LSTM model, claims that it “has a much less intrusive number of false positives, and focuses specifically on the identification of 3 concepts types – problems, tests, and treatments.”

A lot of the work in NLP in recent years has been in recognizing entities, and methods based on, e.g., Bidirectional Encoder Representations from Transformers (BERT) [28] have advanced the state of the art. However, less work has been done on normalizing entities, and in this work, normalization was key: we wanted to understand the particular disease concepts. Methods such as biomedical named entity recognition and multi-type normalization (BERN) [29] do perform normalization, and this might be a useful area for future evaluation.

## Conclusion

It is difficult for human experts to identify all the concepts in clinical text, unrealistic to expect an NLP engine to handle all linguistic variations in parsing sentences, and impossible to maintain a dictionary with enough synonyms to support easy mapping to all concepts. Nonetheless, the results from this pilot study are encouraging that good performance can be achieved with commercially available systems, especially for disorders and clinical findings. We encourage more researchers to build NLP engines that cast a wide net and that improve upon the results presented in our paper. Doing so will get us closer to building a state-of-the-art system for the extraction and normalization of a broad array of clinical problems. Such a system could populate structured problem lists in conjunction with review by a clinician or appropriately trained abstractor.

## Electronic Supplementary Material

ESM 1 (DOCX 50 kb)

## Data Availability

Not applicable.
