# Supplementary material for: Evaluation of a Concept Mapping Task Using Named Entity Recognition and Normalization in Unstructured Clinical Text
Source: J Healthc Inform Res. 2020 Oct 16;4(4):395–410. doi: 10.1007/s41666-020-00079-z (PMC8982815; doi:10.1007/s41666-020-00079-z)
Supplement: Supplementary file 1 — (DOCX 50 kb) [file 41666_2020_79_MOESM1_ESM.docx]

**Title**

Evaluation of a concept mapping task using named entity recognition and normalization in unstructured clinical text

**Journal**

Journal of Healthcare Informatics Research

**Authors**

Sapna Trivedi^1^, Roger Gildersleeve^2^, Sandra Franco^2^, Andrew S. Kanter^2^, Afzal Chaudhry^1^

**Author information**

1. Department of Clinical Informatics, University of Cambridge, UK

2. Intelligent Medical Objects (IMO), Rosemont, IL

**Corresponding author**

Sapna.trivedi@addenbrookes.nhs.uk

**Online Resource 1. EASL representation of Query**

version: 5.3.1

creationDate: '2018-05-11 15:58:56'

multiquery:

Union: [/api;type=saved_query/__private__/I2EAdmin/problem_procedure_history%20(11).i2qy,

/api;type=saved_query/__private__/I2EAdmin/history_problem_procedure%20(19).i2qy]

output:

outputSettings: {allResults: true, outputOrdering: document}

creator: minnamorati

querySnapshots:

/api;type=saved_query/__private__/I2EAdmin/history_problem_procedure%20(19).i2qy:

version: 5.3.1

creationDate: '2018-05-10 12:48:58'

query:

document:

- phrase:

comment: this item clears variables

expression: |-

(function()

i2e.set('Negation', 'Asserted')

i2e.set('History', '')

return PT

end)()

id: phrase1

maxWordGap: 5

ptSource: expression

showInColumn: false

showInHitColumn: false

of:

- alternative:

expression: i2e.set('Negation', PT)

id: alternative4

label: Negation

ptSource: expression

quantifier: {minimum: 0, maximum: 1}

showInColumn: false

showInHitColumn: false

of:

- phrase:

allowOverlap: true

distinguishHits: false

label: Uncertain

maxWordGap: 5

ptSource: label

of:

- alternative:

view: {layout: list}

of:

- macro:

wordAccentConstraint: 'No'

wordDialectConstraint: 'No'

wordHomoglyphConstraint: 'No'

wordMisspellingConstraint: 'No'

wordOcrConstraint: 'No'

snid: Linguamatics.PREPOS

pt: Possible Pre-Negation

of:

- alternative:

macroPt: Possible Pre-Negation

macroSnid: Linguamatics.PREPOS

of:

- considering

- phrase: [consider, adding, to]

- probable

- consider

- whether

- maybe

- discussed

- possible

- phrase: [suggestive, of]

- doubtful

- suggests

- likely

- unlikely

- phrase: [not, likely]

- potential

- phrase: [consider, adjusting]

- suspected

- phrase: [test, for]

- phrase: [suspicious, for]

- discussing

- discuss

- if

- ro

- r/o

- phrase: [r, /, o]

- phrase: [what, must, be, ruled, out, is]

- phrase:

- alternative: [can't, cannot, mightn't, shouldn't,

mustn't]

- be

- word: {text: rules, morphoVariants: true}

- out

- phrase:

- alternative: [doesn't, didn't, can't, cannot, mightn't,

shouldn't, mustn't, wouldn't, isn't, wasn't, aren't,

weren't]

- be

- word: {text: rules, morphoVariants: true}

- out

- phrase:

- alternative: [does, did, can, could, may, might,

should, must, would, will, ought, is, was, are,

were]

- not

- word: {text: rules, morphoVariants: true}

- out

- phrase:

- alternative:

- hasn't

- haven't

- hadn't

- phrase:

displayChildren: false

of: [has, not]

- phrase:

displayChildren: false

of: [have, not]

- phrase:

displayChildren: false

of: [had, not]

- word: {text: rules, morphoVariants: true}

- out

- likelihood

- word: {text: suggest, morphoVariants: true}

- phrase:

displayChildren: false

of: [Question, of]

- word: {text: likely, morphoVariants: true}

- if

- phrase:

displayChildren: false

quantifier: {minimum: 0, maximum: 0}

of:

- alternative:

view: {layout: list}

of:

- due

- word: {text: caused, morphoVariants: true}

- because

- word: {text: result, label: Dont use morph here. Needs

to hit "result of" only}

- secondary

- word: {text: source, morphoVariants: true}

- {class: /prep}

- word:

text: ':'

quantifier: {minimum: 0, maximum: 0}

- phrase:

allowOverlap: true

label: Negated

maxWordGap: 5

ptSource: label

of:

- phrase:

label: Negated

ptSource: label

of:

- alternative:

quantifier: {minimum: 0, maximum: 0}

of: [if]

- macro:

wordAccentConstraint: 'No'

wordDialectConstraint: 'No'

wordHomoglyphConstraint: 'No'

wordMisspellingConstraint: 'No'

wordOcrConstraint: 'No'

snid: Linguamatics.PREN

pt: Pre-Negation

of:

- alternative:

macroPt: Pre-Negation

macroSnid: Linguamatics.PREN

of:

- phrase:

- word: {text: do, morphoVariants: true}

- not

- reveal

- phrase:

- alternative:

- adequate

- phrase:

displayChildren: false

of: [adequate, to]

- adequately

- sufficient

- phrase:

displayChildren: false

of: [sufficient, to]

- sufficiently

- word: {text: rules, morphoVariants: true}

- alternative:

- him

- her

- patient

- pt

- phrase:

displayChildren: false

of: [the, patient]

- phrase:

displayChildren: false

of: [the, pt]

- out

- alternative: [for, against]

- phrase:

- alternative:

- adequate

- phrase:

displayChildren: false

of: [adequate, to]

- adequately

- sufficient

- phrase:

displayChildren: false

of: [sufficient, to]

- sufficiently

- be

- word: {text: rules, morphoVariants: true}

- alternative:

- him

- her

- patient

- pt

- phrase:

displayChildren: false

of: [the, patient]

- phrase:

displayChildren: false

of: [the, pt]

- out

- alternative: [for, against]

- phrase:

- alternative:

- adequate

- phrase:

displayChildren: false

of: [adequate, to]

- adequately

- sufficient

- phrase:

displayChildren: false

of: [sufficient, to]

- sufficiently

- word: {text: rules, morphoVariants: true}

- out

- alternative:

- him

- her

- patient

- pt

- phrase:

displayChildren: false

of: [the, patient]

- phrase:

displayChildren: false

of: [the, pt]

- phrase:

- alternative:

- adequate

- phrase:

displayChildren: false

of: [adequate, to]

- adequately

- sufficient

- phrase:

displayChildren: false

of: [sufficient, to]

- sufficiently

- word: {text: rules, morphoVariants: true}

- out

- phrase:

- alternative:

- adequate

- phrase:

displayChildren: false

of: [adequate, to]

- adequately

- sufficient

- phrase:

displayChildren: false

of: [sufficient, to]

- sufficiently

- be

- word: {text: rules, morphoVariants: true}

- out

- phrase: [risk, of]

- phrase: [not, associated, with]

- resolved

- word: {text: discontinue, morphoVariants: true}

- cannot

- phrase: [negative, for]

- phrase: ['no', suggestion, of]

- not

- phrase: [ruled, out]

- phrase:

- alternative:

- does

- did

- is

- was

- are

- were

- phrase:

displayChildren: false

of: [has, been]

- phrase:

displayChildren: false

of: [had, been]

- phrase:

displayChildren: false

of: [have, been]

- has

- had

- have

- word: {text: rules, morphoVariants: true}

- out

- phrase:

- alternative:

- does

- did

- is

- was

- are

- were

- phrase:

displayChildren: false

of: [has, been]

- phrase:

displayChildren: false

of: [had, been]

- phrase:

displayChildren: false

of: [have, been]

- has

- had

- have

- word: {text: rules, morphoVariants: true}

- alternative:

- him

- her

- patient

- pt

- phrase:

displayChildren: false

of: [the, patient]

- phrase:

displayChildren: false

of: [the, pt]

- out

- phrase:

- alternative:

- does

- did

- is

- was

- are

- were

- phrase:

displayChildren: false

of: [has, been]

- phrase:

displayChildren: false

of: [had, been]

- phrase:

displayChildren: false

of: [have, been]

- has

- had

- have

- word: {text: rules, morphoVariants: true}

- out

- alternative:

- him

- her

- patient

- pt

- phrase:

displayChildren: false

of: [the, patient]

- phrase:

displayChildren: false

of: [the, pt]

- phrase:

- alternative: [can, could, may, might]

- be

- word: {text: rules, morphoVariants: true}

- out

- alternative:

- him

- her

- patient

- pt

- phrase:

displayChildren: false

of: [the, patient]

- phrase:

displayChildren: false

of: [the, pt]

- phrase:

- alternative: [can, could, may, might]

- be

- word: {text: rules, morphoVariants: true}

- alternative:

- him

- her

- patient

- pt

- phrase:

displayChildren: false

of: [the, patient]

- phrase:

displayChildren: false

of: [the, pt]

- out

- phrase:

- alternative: [can, could, may, might]

- be

- word: {text: rules, morphoVariants: true}

- out

- phrase:

- ruled

- alternative:

- him

- her

- patient

- pt

- phrase:

displayChildren: false

of: [the, patient]

- phrase:

displayChildren: false

of: [the, pt]

- out

- phrase:

- ruled

- out

- alternative:

- him

- her

- patient

- pt

- phrase:

displayChildren: false

of: [the, patient]

- phrase:

displayChildren: false

of: [the, pt]

- phrase:

- alternative:

- him

- her

- patient

- pt

- phrase:

displayChildren: false

of: [the, patient]

- phrase:

displayChildren: false

of: [the, pt]

- was

- not

- phrase: ['no', suspicious]

- phrase: ['no', evidence, to, suggest]

- word: {text: declines, morphoVariants: true}

- phrase: [not, had]

- phrase: ['no', other, evidence]

- phrase: [test, for]

- phrase: [didn't, want, to]

- declined

- denies

- avoid

- phrase:

- rat

- alternative:

- him

- her

- patient

- pt

- phrase:

displayChildren: false

of: [the, patient]

- phrase:

displayChildren: false

of: [the, pt]

- than

- phrase: [not, exhibit]

- phrase: [checked, for]

- word: {text: -ve}

- denied

- phrase: [evaluate, for]

- phrase: [unwilling, to]

- phrase: ['no', findings, to, indicate]

- phrase:

- 'no'

- alternative:

- sign

- evidence

- suggestion

- appearance

- symptom

- phrase:

displayChildren: false

of: [clinical, symptom]

- phrase:

displayChildren: false

of: [other, symptom]

- of

- phrase:

- alternative:

- without

- wo

- w/o

- w/out

- phrase:

displayChildren: false

of: [w, /, o]

- phrase:

displayChildren: false

of: [with, out]

- phrase:

displayChildren: false

of: [w, out]

- phrase:

displayChildren: false

of: [w, /, out]

- sign

- of

- phrase: [eval, for]

- phrase: ['no', new]

- phrase: ['no', cause, of]

- phrase: [not, have]

- discontinued

- phrase:

- what

- must

- be

- word: {text: rules, morphoVariants: true}

- out

- is

- phrase:

- word: {text: rules, morphoVariants: true}

- the

- patient

- out

- against

- phrase: ['no', new, evidence]

- phrase: ['no', significant]

- phrase: [not, know, of]

- phrase: [free, of]

- phrase: ['no', complaints, of]

- phrase:

- alternative:

- without

- wo

- w/o

- w/out

- phrase:

displayChildren: false

of: [w, /, o]

- phrase:

displayChildren: false

of: [with, out]

- phrase:

displayChildren: false

of: [w, out]

- phrase:

displayChildren: false

of: [w, /, out]

- any

- evidence

- of

- phrase:

- alternative:

- without

- wo

- w/o

- w/out

- phrase:

displayChildren: false

of: [w, /, o]

- phrase:

displayChildren: false

of: [with, out]

- phrase:

displayChildren: false

of: [w, out]

- phrase:

displayChildren: false

of: [w, /, out]

- indication

- of

- phrase:

- adequate

- to

- word: {text: rules, morphoVariants: true}

- alternative:

- him

- her

- patient

- pt

- phrase:

displayChildren: false

of: [the, patient]

- phrase:

displayChildren: false

of: [the, pt]

- out

- for

- phrase: ['no', radiographic, evidence, of]

- phrase: [not, appear]

- phrase:

- alternative:

- without

- wo

- w/o

- w/out

- phrase:

displayChildren: false

of: [w, /, o]

- phrase:

displayChildren: false

of: [with, out]

- phrase:

displayChildren: false

of: [w, out]

- phrase:

displayChildren: false

of: [w, /, out]

- evidence

- phrase: [never, had]

- phrase: [not, see]

- phrase: [fails, to, reveal]

- phrase: [to, exclude]

- excluding

- phrase: [unremarkable, for]

- phrase: [not, feel]

- phrase: [not, appreciate]

- phrase: [not, complain, of]

- phrase: [not, demonstrate]

- phrase: [not, to, be]

- phrase: [not, a, candidate, for]

- phrase:

- be

- word: {text: rules, morphoVariants: true}

- out

- for

- phrase: [absence, of]

- phrase: ['no', mammographic, evidence, of]

- denying

- phrase: [cannot, see]

- 'no'

- phrase: [with, 'no']

- phrase: ['no', signs, of]

- phrase: ['no', findings, of]

- phrase: [not, want, to]

- phrase: ['no', abnormal]

- phrase: [not, known, to, have]

- phrase: ['no', evidence]

- phrase: [never, developed]

- alternative:

- without

- wo

- w/o

- w/out

- phrase:

displayChildren: false

of: [w, /, o]

- phrase:

displayChildren: false

of: [with, out]

- phrase:

displayChildren: false

of: [w, out]

- phrase:

displayChildren: false

of: [w, /, out]

- word:

text: change

quantifier: {minimum: 0, maximum: 0}

- alternative:

quantifier: {minimum: 0, maximum: 0}

view: {layout: list}

of: [':', therefore]

- alternative:

expression: i2e.set('History', PT)

id: alternative2

label: History

ptSource: expression

quantifier: {minimum: 0, maximum: 1}

showInColumn: false

showInHitColumn: false

of:

- phrase:

displayChildren: false

label: Family History

ptSource: label

of:

- link: {path: /api;type=saved_query/problem_only/family_hist.i2qy}

- phrase:

label: History

ptSource: label

of:

- phrase:

allowOverlap: true

label: Patient History

ptSource: label

unordered: true

of:

- macro:

quantifier: {minimum: 0, maximum: 0}

wordAccentConstraint: 'No'

wordDialectConstraint: 'No'

wordHomoglyphConstraint: 'No'

wordMisspellingConstraint: 'No'

wordOcrConstraint: 'No'

snid: Linguamatics.FAMHIST

pt: Family History

of:

- alternative:

macroPt: Family History

macroSnid: Linguamatics.FAMHIST

of:

- phrase: [fam, hx]

- phrase: [family, history]

- father

- paternal

- grandmother

- mum

- dad

- famhx

- maternal

- grandad

- phrase: [fam, hist]

- uncle

- mom

- cousin

- phrase: [family, hist]

- sister

- phrase: [family, hx]

- brother

- grandfather

- aunt

- fhx

- mother

- alternative:

label: Patient History

ptSource: label

view: {layout: list}

of:

- phrase:

displayChildren: false

of: [history]

- hx

- h/o

- phrase:

displayChildren: false

of: [h, /, o]

- ho

- hxo

- phrase:

- 'on'

- class: {snid: date.date, pt: Specified Date}

- post

- phrase:

displayChildren: false

of: [s, /, p]

- s/p

- past

- phrase:

allowOverlap: true

comment: this item assigns negation

expression: i2e.get('Negation')

id: phrase2

ptSource: expression

showInHitColumn: false

of:

- link: {path: /api;type=saved_query/__private__/I2EAdmin/problem_procedure%20(23).i2qy,

id: link1}

- phrase:

comment: this item assigns history

expression: |-

(function()

local pt = i2e.get('CPT')

if string.match(pt, '[Hh]istory[%s%:,;]') or

string.match(pt, '[Pp][ao]st[%s%:,;]') or

string.match(pt, '[Hh]%/[oO][%s%:,;]') or

string.match(pt, '[Ss]%/[Pp][%s%:,;]') or

string.match(pt, '[Hh][Xx][%s%:,;]') then

i2e.set('History', 'History')

end

if string.match(pt, '[Ff]amily [Hh]istory[%s%:,;]') or

string.match(pt, '[Ff]%/[Hh][Xx]?[%s%:,;]') or

string.match(pt, '[Ff][Hh][Xx]?[%s%:,;]') then

i2e.set('History', 'Family History')

end

return i2e.get('History')

end)()

id: phrase3

ptSource: expression

showInHitColumn: false

of:

- class:

snid: /word

where:

- alternative:

label: Noisy

quantifier: {minimum: 0, maximum: 0}

of:

- link: {path: /api;type=saved_query/__private__/I2EAdmin/noisy%20(4).i2qy}

- alternative:

quantifier: {minimum: 0, maximum: 0}

of:

- link: {path: /api;type=saved_query/problem_only/history_postmod.i2qy}

output:

columns:

- {id: alternative2}

- {id: alternative4}

- {id: phrase1}

- {title: Negation, id: phrase2}

- {title: Tense, id: phrase3}

- id: link1.alternative3

itemOptions: {showInHitColumn: false}

linkItemId: link1

- {id: link1.alternative1, linkItemId: link1}

- id: link1.macro2

itemOptions: {showInColumn: false, showInHitColumn: false}

linkItemId: link1

- id: link1.class1

itemOptions:

columnOptions: [NodeID, PT]

showInColumn: false

showInHitColumn: false

linkItemId: link1

- id: link1.alternative5

itemOptions: {showInColumn: false, showInHitColumn: false}

linkItemId: link1

columnLocations: [5, 6, 4, 3, 0, 1, 2, 7, 8, 9]

outputSettings: {allResults: true, allTime: true, boundaries: Exact}

showFilters: true

useOutputEditor: true

filters:

- of: PT

where: [link1.macro2, link1.alternative5]

condition: notEquals

- of: PT

where: [link1.alternative5]

condition: notEquals

creator: minnamorati

/api;type=saved_query/__private__/I2EAdmin/noisy%20(4).i2qy:

version: 5.3.1

creationDate: '2018-08-14 16:20:19'

query:

document:

- alternative:

- word: {text: add, caseSensitive: true}

- word: {text: all, caseSensitive: true}

- word: {text: pat, caseSensitive: true}

- phrase:

displayChildren: false

of: [date, of, birth]

- phrase:

displayChildren: false

of:

- word: {text: '[0-9]{1,2}', allowAccents: false, allowDialect: false,

allowHomoglyphs: false, caseSensitive: true, matchType: Regexp}

- years

- of

- age

- phrase:

- alternative: [activities, activity]

- involving

creator: minnamorati

/api;type=saved_query/__private__/I2EAdmin/noisy%20(5).i2qy:

version: 5.3.1

creationDate: '2018-08-14 16:20:19'

query:

document:

- alternative:

- word: {text: add, caseSensitive: true}

- word: {text: all, caseSensitive: true}

- word: {text: pat, caseSensitive: true}

- phrase:

displayChildren: false

of: [date, of, birth]

- phrase:

displayChildren: false

of:

- word: {text: '[0-9]{1,2}', allowAccents: false, allowDialect: false,

allowHomoglyphs: false, caseSensitive: true, matchType: Regexp}

- years

- of

- age

- phrase:

- alternative: [activities, activity]

- involving

creator: minnamorati

/api;type=saved_query/__private__/I2EAdmin/problem_procedure%20(22).i2qy:

version: 5.3.1

creationDate: '2018-05-11 08:49:05'

query:

document:

- phrase:

ptSource: expression

showInColumn: false

showInHitColumn: false

of:

- phrase:

quantifier: {minimum: 0, maximum: 1}

of:

- macro:

excludeIfBetterMatch: true

wordAccentConstraint: 'No'

wordDialectConstraint: 'No'

wordHomoglyphConstraint: 'No'

wordMisspellingConstraint: 'No'

wordOcrConstraint: 'No'

snid: linguamatics_elu.ELU_list

pt: list

of:

- alternative:

macroPt: list

macroSnid: linguamatics_elu.ELU_list

of:

- {class: /ng}

- phrase:

- {class: /ng}

- word: {text: ',', allowAccents: false, allowDialect: false,

allowHomoglyphs: false}

- {class: /ng}

- phrase:

- {class: /ng}

- word: {text: ',', allowAccents: false, allowDialect: false,

allowHomoglyphs: false}

- {class: /ng}

- word: {text: ',', allowAccents: false, allowDialect: false,

allowHomoglyphs: false}

- {class: /ng}

- phrase:

- {class: /ng}

- word: {text: ',', allowAccents: false, allowDialect: false,

allowHomoglyphs: false}

- {class: /ng}

- word: {text: ',', allowAccents: false, allowDialect: false,

allowHomoglyphs: false}

- {class: /ng}

- word: {text: ',', allowAccents: false, allowDialect: false,

allowHomoglyphs: false}

- {class: /ng}

- phrase:

- {class: /ng}

- word: {text: ',', allowAccents: false, allowDialect: false,

allowHomoglyphs: false}

- {class: /ng}

- word: {text: ',', allowAccents: false, allowDialect: false,

allowHomoglyphs: false}

- {class: /ng}

- word: {text: ',', allowAccents: false, allowDialect: false,

allowHomoglyphs: false}

- {class: /ng}

- word: {text: ',', allowAccents: false, allowDialect: false,

allowHomoglyphs: false}

- {class: /ng}

- macro:

excludeIfBetterMatch: true

quantifier: {minimum: 0, maximum: 1}

wordAccentConstraint: 'No'

wordDialectConstraint: 'No'

wordHomoglyphConstraint: 'No'

wordMisspellingConstraint: 'No'

wordOcrConstraint: 'No'

snid: linguamatics_elu.ELU_list_connector_last

pt: list connector last

of:

- alternative:

macroPt: list connector last

macroSnid: linguamatics_elu.ELU_list_connector_last

of:

- alternative:

- word: {text: and, allowAccents: false, allowDialect: false,

allowHomoglyphs: false}

- word: {text: or, allowAccents: false, allowDialect: false,

allowHomoglyphs: false}

- phrase:

displayChildren: false

of:

- word: {text: and, allowAccents: false, allowDialect: false,

allowHomoglyphs: false}

- word: {text: /, allowAccents: false, allowDialect: false,

allowHomoglyphs: false}

- word: {text: or, allowAccents: false, allowDialect: false,

allowHomoglyphs: false}

- phrase:

- word: {text: ',', allowAccents: false, allowDialect: false,

allowHomoglyphs: false}

- word: {text: and, allowAccents: false, allowDialect: false,

allowHomoglyphs: false}

- phrase:

- word: {text: ',', allowAccents: false, allowDialect: false,

allowHomoglyphs: false}

- word: {text: or, allowAccents: false, allowDialect: false,

allowHomoglyphs: false}

- phrase:

- word: {text: ',', allowAccents: false, allowDialect: false,

allowHomoglyphs: false}

- word: {text: and, allowAccents: false, allowDialect: false,

allowHomoglyphs: false}

- word: {text: /, allowAccents: false, allowDialect: false,

allowHomoglyphs: false}

- word: {text: or, allowAccents: false, allowDialect: false,

allowHomoglyphs: false}

- phrase:

allowOverlap: true

unordered: true

of:

- alternative:

expression: i2e.get('Domain')

id: alternative3

label: Domain

ptSource: expression

showInHitColumn: false

of:

- alternative:

comment: this item returns the text of the hit to ensure it

does not match that of the blacklist.

expression: Text

id: alternative5

label: FilterBlacklist

ptSource: expression

showInColumn: false

showInHitColumn: false

of:

- alternative:

columnOptions: [NodeID, PT]

expression: i2e.set('CPT', PT)

id: alternative1

label: IMO Concept

ptSource: expression

of:

- class:

columnOptions: [NodeID, PT]

expression: |-

(function()

i2e.set('text', Text)

return i2e.set('Domain', 'Problem', PT)

end)()

id: class1

includeSpellingCorrections: true

ptSource: expression

showInColumn: false

showInHitColumn: false

snid: 1.PROBLEM

where:

- phrase:

quantifier: {minimum: 0, maximum: 1}

unordered: true

of:

- macro:

excludeIfBetterMatch: true

expression: Text

id: macro2

ptSource: expression

showInColumn: false

showInHitColumn: false

snid: imo.diseases

pt: Diseases

of:

- alternative:

macroPt: Diseases

macroSnid: imo.diseases

of:

- phrase: [24-year-old, age]

- phrase: [able, to, see]

- accident

- accidents

- phrase: [accident, to]

- activities

- activity

- add

- afraid

- aftercare

- phrase: [after, treatment]

- allergic

- allergies

- allergy

- american-european

- phrase: [a, mass]

- analgesia

- angry

- asleep

- assault

- assaulted

- phrase: [assault, by]

- asymptomatic

- audiology

- awake

- birth

- phrase: [blood, transfusion]

- phrase: [body, image]

- phrase: [breath, sounds]

- phrase: [brief, hospital, course]

- bulging

- bump

- Bump

- phrase: [check, up]

- phrase: [chief, complaint]

- childhood

- clear-cut

- cold

- cold-knife

- collapse

- colon

- colonic

- co-morbidities

- complaint

- complaints

- complications

- consultation

- corn

- counseling

- counselling

- phrase: [counseling, service]

- phrase: [counselling, service]

- counselor

- counsellor

- cut

- cuts

- cutting

- cyst

- cysts

- death

- depressed

- phrase: [diagnostic, test]

- phrase: [diagnostic, tests]

- died

- diet

- diets

- phrase: [discharge, status]

- discomfort

- disease

- diseases

- dislocation

- disorder

- diving

- double-blind

- phrase: [drug, effect]

- phrase: [drug, effects]

- dialysis

- endocrine

- ESR

- european

- phrase: [european, ancestry]

- event

- exam

- examination

- examine

- examined

- excitement

- phrase: [eyes, closed]

- fall

- falls

- falling

- fat

- female

- fistula

- floppy

- flush

- phrase: [flu, shot]

- phrase: [follow, up]

- follow-up

- word: {text: -follow-up}

- phrase: [follow-up, visit]

- phrase: [follow, up, visit]

- phrase: [follow, up, exam]

- phrase: [follow-up, exam]

- phrase: [follow, up, examination]

- phrase: [follow-up, examination]

- fracture

- fractured

- frozen

- gases

- phrase: [general, physical, examination]

- phrase: [generally, unwell]

- genitourinary

- gift

- golf

- graft

- grafts

- phrase: [heart, sounds]

- hemodialysis

- phrase: [her, disease]

- phrase: [history, of, present, illness]

- phrase: [home, oxygen]

- hospice

- ill

- illness

- impairment

- injury

- injuries

- investigator-blind

- jumping

- kayaking

- phrase: [laboratory, procedures]

- lac

- lifestyle

- phrase: [life, style]

- low-fat

- lump

- lumps

- phrase: [lying, in, bed]

- lyme

- mass

- mass+

- phrase: [medical, problem]

- phrase: [medical, problems]

- mornings

- NEC

- nervous

- nonverbal

- numb

- numbness

- phrase: [occasional, pain]

- phrase: [occupational, therapy]

- phrase: [old, age]

- old-age

- om

- OM

- OM-1

- OM-2

- phrase: [other, problem]

- overlying

- pacemaker

- pain

- pains

- pat

- phrase: [physical, exam]

- phrase: [physical, examination]

- phrase: [physical, therapy]

- physiotherapy

- plague

- phrase: [plastic, surgery]

- phrase: [peritoneal, dialysis]

- positive-pressure

- posttreatment

- pressure

- pressure-controlled

- pressure-targeted

- problem

- 'problem:'

- problems

- phrase: [pulmonary, function, tests]

- puncture

- rafting

- phrase: [rca, stent]

- phrase: [reason, for, consult]

- phrase: [reason, for, consultation]

- redness

- rehabilitaion

- rehabilitation

- right-dominant

- phrase: [right, dominant]

- rigid

- rocking

- rowing

- running

- sating

- scar

- scars

- screening

- screenings

- phrase: [screening, for]

- sensitivity

- phrase: [sensitive, stomach]

- separation

- sequela

- shock

- short

- short-term

- phrase: [short, term]

- sick

- sign

- signs

- skiing

- phrase: [skin, care]

- sleeping

- smith

- sounds

- phrase: [special, circumstances]

- stone

- stress

- suffer

- suffering

- suffers

- phrase: [surgery, history]

- phrase: [surgical, history]

- phrase: [surgical, procedure]

- suspicion

- suspicious

- swelling

- swimming

- swollen

- syndrome

- syndromes

- tearing

- tears

- tender

- tenderness

- tennis

- therapeutic

- therapeutics

- therapy

- therapies

- throwing

- tingling

- tired

- transfusion

- transplant

- trauma

- traumas

- travel

- travels

- travelling

- treat

- treated

- treatment

- treatments

- phrase: [treatment, option]

- phrase: [treatment, options]

- twin

- ulceration

- phrase: [unable, to, see]

- vacuuming

- vibration

- walked

- walks

- weak

- weakness

- phrase: [well, child]

- well-child

- whitaker

- wind

- withdrawal

- phrase: [work, out]

- worry

- worried

- worries

- worrying

- wound

- wounds

- 'wounds:'

- macro:

quantifier: {minimum: 0, maximum: 0}

snid: imo.diseases

pt: Diseases

of:

- alternative:

macroPt: Diseases

macroSnid: imo.diseases

of:

- phrase: [24-year-old, age]

- phrase: [able, to, see]

- accident

- accidents

- phrase: [accident, to]

- activities

- activity

- add

- afraid

- aftercare

- phrase: [after, treatment]

- allergic

- allergies

- allergy

- american-european

- phrase: [a, mass]

- analgesia

- angry

- asleep

- assault

- assaulted

- phrase: [assault, by]

- asymptomatic

- audiology

- awake

- birth

- phrase: [blood, transfusion]

- phrase: [body, image]

- phrase: [breath, sounds]

- phrase: [brief, hospital, course]

- bulging

- bump

- Bump

- phrase: [check, up]

- phrase: [chief, complaint]

- childhood

- clear-cut

- cold

- cold-knife

- collapse

- colon

- colonic

- co-morbidities

- complaint

- complaints

- complications

- consultation

- corn

- counseling

- counselling

- phrase: [counseling, service]

- phrase: [counselling, service]

- counselor

- counsellor

- cut

- cuts

- cutting

- cyst

- cysts

- death

- depressed

- phrase: [diagnostic, test]

- phrase: [diagnostic, tests]

- died

- diet

- diets

- phrase: [discharge, status]

- discomfort

- disease

- diseases

- dislocation

- disorder

- diving

- double-blind

- phrase: [drug, effect]

- phrase: [drug, effects]

- dialysis

- endocrine

- ESR

- european

- phrase: [european, ancestry]

- event

- exam

- examination

- examine

- examined

- excitement

- phrase: [eyes, closed]

- fall

- falls

- falling

- fat

- female

- fistula

- floppy

- flush

- phrase: [flu, shot]

- phrase: [follow, up]

- follow-up

- word: {text: -follow-up}

- phrase: [follow-up, visit]

- phrase: [follow, up, visit]

- phrase: [follow, up, exam]

- phrase: [follow-up, exam]

- phrase: [follow, up, examination]

- phrase: [follow-up, examination]

- fracture

- fractured

- frozen

- gases

- phrase: [general, physical, examination]

- phrase: [generally, unwell]

- genitourinary

- gift

- golf

- graft

- grafts

- phrase: [heart, sounds]

- hemodialysis

- phrase: [her, disease]

- phrase: [history, of, present, illness]

- phrase: [home, oxygen]

- hospice

- ill

- illness

- impairment

- injury

- injuries

- investigator-blind

- jumping

- kayaking

- phrase: [laboratory, procedures]

- lac

- lifestyle

- phrase: [life, style]

- low-fat

- lump

- lumps

- phrase: [lying, in, bed]

- lyme

- mass

- mass+

- phrase: [medical, problem]

- phrase: [medical, problems]

- mornings

- NEC

- nervous

- nonverbal

- numb

- numbness

- phrase: [occasional, pain]

- phrase: [occupational, therapy]

- phrase: [old, age]

- old-age

- om

- OM

- OM-1

- OM-2

- phrase: [other, problem]

- overlying

- pacemaker

- pain

- pains

- pat

- phrase: [physical, exam]

- phrase: [physical, examination]

- phrase: [physical, therapy]

- physiotherapy

- plague

- phrase: [plastic, surgery]

- phrase: [peritoneal, dialysis]

- positive-pressure

- posttreatment

- pressure

- pressure-controlled

- pressure-targeted

- problem

- 'problem:'

- problems

- phrase: [pulmonary, function, tests]

- puncture

- rafting

- phrase: [rca, stent]

- phrase: [reason, for, consult]

- phrase: [reason, for, consultation]

- redness

- rehabilitaion

- rehabilitation

- right-dominant

- phrase: [right, dominant]

- rigid

- rocking

- rowing

- running

- sating

- scar

- scars

- screening

- screenings

- phrase: [screening, for]

- sensitivity

- phrase: [sensitive, stomach]

- separation

- sequela

- shock

- short

- short-term

- phrase: [short, term]

- sick

- sign

- signs

- skiing

- phrase: [skin, care]

- sleeping

- smith

- sounds

- phrase: [special, circumstances]

- stone

- stress

- suffer

- suffering

- suffers

- phrase: [surgery, history]

- phrase: [surgical, history]

- phrase: [surgical, procedure]

- suspicion

- suspicious

- swelling

- swimming

- swollen

- syndrome

- syndromes

- tearing

- tears

- tender

- tenderness

- tennis

- therapeutic

- therapeutics

- therapy

- therapies

- throwing

- tingling

- tired

- transfusion

- transplant

- trauma

- traumas

- travel

- travels

- travelling

- treat

- treated

- treatment

- treatments

- phrase: [treatment, option]

- phrase: [treatment, options]

- twin

- ulceration

- phrase: [unable, to, see]

- vacuuming

- vibration

- walked

- walks

- weak

- weakness

- phrase: [well, child]

- well-child

- whitaker

- wind

- withdrawal

- phrase: [work, out]

- worry

- worried

- worries

- worrying

- wound

- wounds

- 'wounds:'

- alternative:

label: Noisy

quantifier: {minimum: 0, maximum: 0}

of:

- link: {path: /api;type=saved_query/__private__/I2EAdmin/noisy%20(4).i2qy}

output:

columns:

- {id: macro2}

- {id: class1}

- {id: alternative1}

- {id: alternative5}

- {id: alternative3}

columnLocations: [4, 2, 0, 1, 3]

outputSettings: {boundaries: Exact}

showFilters: true

useOutputEditor: true

filters:

- of: PT

where: [macro2, alternative5]

condition: notEquals

- of: PT

where: [alternative5]

condition: notEquals

creator: minnamorati

/api;type=saved_query/__private__/I2EAdmin/problem_procedure%20(23).i2qy:

version: 5.3.1

creationDate: '2018-05-11 08:49:05'

query:

document:

- phrase:

ptSource: expression

showInColumn: false

showInHitColumn: false

of:

- phrase:

quantifier: {minimum: 0, maximum: 1}

of:

- macro:

excludeIfBetterMatch: true

wordAccentConstraint: 'No'

wordDialectConstraint: 'No'

wordHomoglyphConstraint: 'No'

wordMisspellingConstraint: 'No'

wordOcrConstraint: 'No'

snid: linguamatics_elu.ELU_list

pt: list

of:

- alternative:

macroPt: list

macroSnid: linguamatics_elu.ELU_list

of:

- {class: /ng}

- phrase:

- {class: /ng}

- word: {text: ',', allowAccents: false, allowDialect: false,

allowHomoglyphs: false}

- {class: /ng}

- phrase:

- {class: /ng}

- word: {text: ',', allowAccents: false, allowDialect: false,

allowHomoglyphs: false}

- {class: /ng}

- word: {text: ',', allowAccents: false, allowDialect: false,

allowHomoglyphs: false}

- {class: /ng}

- phrase:

- {class: /ng}

- word: {text: ',', allowAccents: false, allowDialect: false,

allowHomoglyphs: false}

- {class: /ng}

- word: {text: ',', allowAccents: false, allowDialect: false,

allowHomoglyphs: false}

- {class: /ng}

- word: {text: ',', allowAccents: false, allowDialect: false,

allowHomoglyphs: false}

- {class: /ng}

- phrase:

- {class: /ng}

- word: {text: ',', allowAccents: false, allowDialect: false,

allowHomoglyphs: false}

- {class: /ng}

- word: {text: ',', allowAccents: false, allowDialect: false,

allowHomoglyphs: false}

- {class: /ng}

- word: {text: ',', allowAccents: false, allowDialect: false,

allowHomoglyphs: false}

- {class: /ng}

- word: {text: ',', allowAccents: false, allowDialect: false,

allowHomoglyphs: false}

- {class: /ng}

- macro:

excludeIfBetterMatch: true

quantifier: {minimum: 0, maximum: 1}

wordAccentConstraint: 'No'

wordDialectConstraint: 'No'

wordHomoglyphConstraint: 'No'

wordMisspellingConstraint: 'No'

wordOcrConstraint: 'No'

snid: linguamatics_elu.ELU_list_connector_last

pt: list connector last

of:

- alternative:

macroPt: list connector last

macroSnid: linguamatics_elu.ELU_list_connector_last

of:

- alternative:

- word: {text: and, allowAccents: false, allowDialect: false,

allowHomoglyphs: false}

- word: {text: or, allowAccents: false, allowDialect: false,

allowHomoglyphs: false}

- phrase:

displayChildren: false

of:

- word: {text: and, allowAccents: false, allowDialect: false,

allowHomoglyphs: false}

- word: {text: /, allowAccents: false, allowDialect: false,

allowHomoglyphs: false}

- word: {text: or, allowAccents: false, allowDialect: false,

allowHomoglyphs: false}

- phrase:

- word: {text: ',', allowAccents: false, allowDialect: false,

allowHomoglyphs: false}

- word: {text: and, allowAccents: false, allowDialect: false,

allowHomoglyphs: false}

- phrase:

- word: {text: ',', allowAccents: false, allowDialect: false,

allowHomoglyphs: false}

- word: {text: or, allowAccents: false, allowDialect: false,

allowHomoglyphs: false}

- phrase:

- word: {text: ',', allowAccents: false, allowDialect: false,

allowHomoglyphs: false}

- word: {text: and, allowAccents: false, allowDialect: false,

allowHomoglyphs: false}

- word: {text: /, allowAccents: false, allowDialect: false,

allowHomoglyphs: false}

- word: {text: or, allowAccents: false, allowDialect: false,

allowHomoglyphs: false}

- phrase:

allowOverlap: true

unordered: true

of:

- alternative:

expression: i2e.get('Domain')

id: alternative3

label: Domain

ptSource: expression

showInHitColumn: false

of:

- alternative:

comment: this item returns the text of the hit to ensure it

does not match that of the blacklist.

expression: Text

id: alternative5

label: FilterBlacklist

ptSource: expression

showInColumn: false

showInHitColumn: false

of:

- alternative:

columnOptions: [NodeID, PT]

expression: i2e.set('CPT', PT)

id: alternative1

label: IMO Concept

ptSource: expression

of:

- class:

columnOptions: [NodeID, PT]

expression: |-

(function()

i2e.set('text', Text)

return i2e.set('Domain', 'Problem', PT)

end)()

id: class1

includeSpellingCorrections: true

ptSource: expression

showInColumn: false

showInHitColumn: false

snid: 1.PROBLEM

where:

- phrase:

quantifier: {minimum: 0, maximum: 1}

unordered: true

of:

- macro:

excludeIfBetterMatch: true

expression: Text

id: macro2

ptSource: expression

showInColumn: false

showInHitColumn: false

snid: imo.diseases

pt: Diseases

of:

- alternative:

macroPt: Diseases

macroSnid: imo.diseases

of:

- phrase: [24-year-old, age]

- phrase: [able, to, see]

- accident

- accidents

- phrase: [accident, to]

- activities

- activity

- add

- afraid

- aftercare

- phrase: [after, treatment]

- allergic

- allergies

- allergy

- american-european

- phrase: [a, mass]

- analgesia

- angry

- asleep

- assault

- assaulted

- phrase: [assault, by]

- asymptomatic

- audiology

- awake

- birth

- phrase: [blood, transfusion]

- phrase: [body, image]

- phrase: [breath, sounds]

- phrase: [brief, hospital, course]

- bulging

- bump

- Bump

- phrase: [check, up]

- phrase: [chief, complaint]

- childhood

- clear-cut

- cold

- cold-knife

- collapse

- colon

- colonic

- co-morbidities

- complaint

- complaints

- complications

- consultation

- corn

- counseling

- counselling

- phrase: [counseling, service]

- phrase: [counselling, service]

- counselor

- counsellor

- cut

- cuts

- cutting

- cyst

- cysts

- death

- depressed

- phrase: [diagnostic, test]

- phrase: [diagnostic, tests]

- died

- diet

- diets

- phrase: [discharge, status]

- discomfort

- disease

- diseases

- dislocation

- disorder

- diving

- double-blind

- phrase: [drug, effect]

- phrase: [drug, effects]

- dialysis

- endocrine

- ESR

- european

- phrase: [european, ancestry]

- event

- exam

- examination

- examine

- examined

- excitement

- phrase: [eyes, closed]

- fall

- falls

- falling

- fat

- female

- fistula

- floppy

- flush

- phrase: [flu, shot]

- phrase: [follow, up]

- follow-up

- word: {text: -follow-up}

- phrase: [follow-up, visit]

- phrase: [follow, up, visit]

- phrase: [follow, up, exam]

- phrase: [follow-up, exam]

- phrase: [follow, up, examination]

- phrase: [follow-up, examination]

- fracture

- fractured

- frozen

- gases

- phrase: [general, physical, examination]

- phrase: [generally, unwell]

- genitourinary

- gift

- golf

- graft

- grafts

- phrase: [heart, sounds]

- hemodialysis

- phrase: [her, disease]

- phrase: [history, of, present, illness]

- phrase: [home, oxygen]

- hospice

- ill

- illness

- impairment

- injury

- injuries

- investigator-blind

- jumping

- kayaking

- phrase: [laboratory, procedures]

- lac

- lifestyle

- phrase: [life, style]

- low-fat

- lump

- lumps

- phrase: [lying, in, bed]

- lyme

- mass

- mass+

- phrase: [medical, problem]

- phrase: [medical, problems]

- mornings

- NEC

- nervous

- nonverbal

- numb

- numbness

- phrase: [occasional, pain]

- phrase: [occupational, therapy]

- phrase: [old, age]

- old-age

- om

- OM

- OM-1

- OM-2

- phrase: [other, problem]

- overlying

- pacemaker

- pain

- pains

- pat

- phrase: [physical, exam]

- phrase: [physical, examination]

- phrase: [physical, therapy]

- physiotherapy

- plague

- phrase: [plastic, surgery]

- phrase: [peritoneal, dialysis]

- positive-pressure

- posttreatment

- pressure

- pressure-controlled

- pressure-targeted

- problem

- 'problem:'

- problems

- phrase: [pulmonary, function, tests]

- puncture

- rafting

- phrase: [rca, stent]

- phrase: [reason, for, consult]

- phrase: [reason, for, consultation]

- redness

- rehabilitaion

- rehabilitation

- right-dominant

- phrase: [right, dominant]

- rigid

- rocking

- rowing

- running

- sating

- scar

- scars

- screening

- screenings

- phrase: [screening, for]

- sensitivity

- phrase: [sensitive, stomach]

- separation

- sequela

- shock

- short

- short-term

- phrase: [short, term]

- sick

- sign

- signs

- skiing

- phrase: [skin, care]

- sleeping

- smith

- sounds

- phrase: [special, circumstances]

- stone

- stress

- suffer

- suffering

- suffers

- phrase: [surgery, history]

- phrase: [surgical, history]

- phrase: [surgical, procedure]

- suspicion

- suspicious

- swelling

- swimming

- swollen

- syndrome

- syndromes

- tearing

- tears

- tender

- tenderness

- tennis

- therapeutic

- therapeutics

- therapy

- therapies

- throwing

- tingling

- tired

- transfusion

- transplant

- trauma

- traumas

- travel

- travels

- travelling

- treat

- treated

- treatment

- treatments

- phrase: [treatment, option]

- phrase: [treatment, options]

- twin

- ulceration

- phrase: [unable, to, see]

- vacuuming

- vibration

- walked

- walks

- weak

- weakness

- phrase: [well, child]

- well-child

- whitaker

- wind

- withdrawal

- phrase: [work, out]

- worry

- worried

- worries

- worrying

- wound

- wounds

- 'wounds:'

- macro:

quantifier: {minimum: 0, maximum: 0}

snid: imo.diseases

pt: Diseases

of:

- alternative:

macroPt: Diseases

macroSnid: imo.diseases

of:

- phrase: [24-year-old, age]

- phrase: [able, to, see]

- accident

- accidents

- phrase: [accident, to]

- activities

- activity

- add

- afraid

- aftercare

- phrase: [after, treatment]

- allergic

- allergies

- allergy

- american-european

- phrase: [a, mass]

- analgesia

- angry

- asleep

- assault

- assaulted

- phrase: [assault, by]

- asymptomatic

- audiology

- awake

- birth

- phrase: [blood, transfusion]

- phrase: [body, image]

- phrase: [breath, sounds]

- phrase: [brief, hospital, course]

- bulging

- bump

- Bump

- phrase: [check, up]

- phrase: [chief, complaint]

- childhood

- clear-cut

- cold

- cold-knife

- collapse

- colon

- colonic

- co-morbidities

- complaint

- complaints

- complications

- consultation

- corn

- counseling

- counselling

- phrase: [counseling, service]

- phrase: [counselling, service]

- counselor

- counsellor

- cut

- cuts

- cutting

- cyst

- cysts

- death

- depressed

- phrase: [diagnostic, test]

- phrase: [diagnostic, tests]

- died

- diet

- diets

- phrase: [discharge, status]

- discomfort

- disease

- diseases

- dislocation

- disorder

- diving

- double-blind

- phrase: [drug, effect]

- phrase: [drug, effects]

- dialysis

- endocrine

- ESR

- european

- phrase: [european, ancestry]

- event

- exam

- examination

- examine

- examined

- excitement

- phrase: [eyes, closed]

- fall

- falls

- falling

- fat

- female

- fistula

- floppy

- flush

- phrase: [flu, shot]

- phrase: [follow, up]

- follow-up

- word: {text: -follow-up}

- phrase: [follow-up, visit]

- phrase: [follow, up, visit]

- phrase: [follow, up, exam]

- phrase: [follow-up, exam]

- phrase: [follow, up, examination]

- phrase: [follow-up, examination]

- fracture

- fractured

- frozen

- gases

- phrase: [general, physical, examination]

- phrase: [generally, unwell]

- genitourinary

- gift

- golf

- graft

- grafts

- phrase: [heart, sounds]

- hemodialysis

- phrase: [her, disease]

- phrase: [history, of, present, illness]

- phrase: [home, oxygen]

- hospice

- ill

- illness

- impairment

- injury

- injuries

- investigator-blind

- jumping

- kayaking

- phrase: [laboratory, procedures]

- lac

- lifestyle

- phrase: [life, style]

- low-fat

- lump

- lumps

- phrase: [lying, in, bed]

- lyme

- mass

- mass+

- phrase: [medical, problem]

- phrase: [medical, problems]

- mornings

- NEC

- nervous

- nonverbal

- numb

- numbness

- phrase: [occasional, pain]

- phrase: [occupational, therapy]

- phrase: [old, age]

- old-age

- om

- OM

- OM-1

- OM-2

- phrase: [other, problem]

- overlying

- pacemaker

- pain

- pains

- pat

- phrase: [physical, exam]

- phrase: [physical, examination]

- phrase: [physical, therapy]

- physiotherapy

- plague

- phrase: [plastic, surgery]

- phrase: [peritoneal, dialysis]

- positive-pressure

- posttreatment

- pressure

- pressure-controlled

- pressure-targeted

- problem

- 'problem:'

- problems

- phrase: [pulmonary, function, tests]

- puncture

- rafting

- phrase: [rca, stent]

- phrase: [reason, for, consult]

- phrase: [reason, for, consultation]

- redness

- rehabilitaion

- rehabilitation

- right-dominant

- phrase: [right, dominant]

- rigid

- rocking

- rowing

- running

- sating

- scar

- scars

- screening

- screenings

- phrase: [screening, for]

- sensitivity

- phrase: [sensitive, stomach]

- separation

- sequela

- shock

- short

- short-term

- phrase: [short, term]

- sick

- sign

- signs

- skiing

- phrase: [skin, care]

- sleeping

- smith

- sounds

- phrase: [special, circumstances]

- stone

- stress

- suffer

- suffering

- suffers

- phrase: [surgery, history]

- phrase: [surgical, history]

- phrase: [surgical, procedure]

- suspicion

- suspicious

- swelling

- swimming

- swollen

- syndrome

- syndromes

- tearing

- tears

- tender

- tenderness

- tennis

- therapeutic

- therapeutics

- therapy

- therapies

- throwing

- tingling

- tired

- transfusion

- transplant

- trauma

- traumas

- travel

- travels

- travelling

- treat

- treated

- treatment

- treatments

- phrase: [treatment, option]

- phrase: [treatment, options]

- twin

- ulceration

- phrase: [unable, to, see]

- vacuuming

- vibration

- walked

- walks

- weak

- weakness

- phrase: [well, child]

- well-child

- whitaker

- wind

- withdrawal

- phrase: [work, out]

- worry

- worried

- worries

- worrying

- wound

- wounds

- 'wounds:'

- alternative:

label: Noisy

quantifier: {minimum: 0, maximum: 0}

of:

- link: {path: /api;type=saved_query/__private__/I2EAdmin/noisy%20(5).i2qy}

output:

columns:

- {id: macro2}

- {id: class1}

- {id: alternative1}

- {id: alternative5}

- {id: alternative3}

columnLocations: [4, 2, 0, 1, 3]

outputSettings: {boundaries: Exact}

showFilters: true

useOutputEditor: true

filters:

- of: PT

where: [macro2, alternative5]

condition: notEquals

- of: PT

where: [alternative5]

condition: notEquals

creator: minnamorati

/api;type=saved_query/__private__/I2EAdmin/problem_procedure_history%20(11).i2qy:

version: 5.3.1

creationDate: '2018-05-10 12:48:58'

query:

document:

- phrase:

comment: this item clears variables

expression: |-

(function()

i2e.set('Negation', 'Asserted')

i2e.set('History', '')

return PT

end)()

id: phrase5

maxWordGap: 5

ptSource: expression

showInColumn: false

showInHitColumn: false

of:

- phrase:

displayChildren: false

expression: i2e.set('Family', 'Family ')

id: phrase3

label: Family History

maxWordGap: 5

ptSource: expression

quantifier: {minimum: 0, maximum: 1}

showInColumn: false

showInHitColumn: false

of:

- link: {path: /api;type=saved_query/problem_only/family_hist.i2qy}

- link: {path: /api;type=saved_query/__private__/I2EAdmin/problem_procedure%20(22).i2qy,

id: link1}

- phrase:

allowOverlap: true

of:

- alternative:

expression: |-

(function()

fam = i2e.get('Family', '')

i2e.set('Family', '')

return i2e.set('History', fam .. 'History')

end)()

id: alternative1

ptSource: expression

showInColumn: false

showInHitColumn: false

of:

- link: {path: /api;type=saved_query/problem_only/history_postmod.i2qy}

- phrase:

comment: this item assigns history

expression: i2e.get('History')

id: phrase6

ptSource: expression

showInHitColumn: false

of:

- phrase:

comment: this item assigns negated

expression: i2e.get('Negation')

id: phrase4

label: Negation

ptSource: expression

showInHitColumn: false

of:

- class:

snid: /word

where:

- alternative:

quantifier: {minimum: 0, maximum: 0}

view: {layout: list}

of:

- word: {text: today, morphoVariants: true}

- word: {text: yesterday, morphoVariants: true}

- word: {text: week, morphoVariants: true}

- word: {text: fortnight, morphoVariants: true}

output:

columns:

- id: link1.alternative3

itemOptions: {showInHitColumn: false}

linkItemId: link1

- {id: link1.alternative1, linkItemId: link1}

- id: link1.macro2

itemOptions: {showInColumn: false, showInHitColumn: false}

linkItemId: link1

- id: link1.class1

itemOptions:

columnOptions: [NodeID, PT]

showInColumn: false

showInHitColumn: false

linkItemId: link1

- id: link1.alternative5

itemOptions: {showInColumn: false, showInHitColumn: false}

linkItemId: link1

- {title: History, id: alternative1}

- {id: phrase3}

- {id: phrase4}

- {id: phrase5}

- {title: Tense, id: phrase6}

columnLocations: [0, 1, 9, 7, 5, 2, 3, 4, 6, 8]

outputSettings: {allResults: true, allTime: true, boundaries: Exact}

showFilters: true

useOutputEditor: true

filters:

- of: PT

where: [link1.macro2, link1.alternative5]

condition: notEquals

- of: PT

where: [link1.alternative5]

condition: notEquals

creator: minnamorati

/api;type=saved_query/problem_only/family_hist.i2qy:

version: 5.3.1

creationDate: '2018-08-31 11:18:21'

query:

document:

- phrase:

maxWordGap: 1

of:

- alternative:

quantifier: {minimum: 0, maximum: 0}

view: {layout: list}

of:

- phrase:

displayChildren: false

of:

- word: {text: live, morphoVariants: true}

- with

- alternative:

label: Family History

ptSource: label

view: {layout: list}

of:

- macro:

wordAccentConstraint: 'No'

wordDialectConstraint: 'No'

wordHomoglyphConstraint: 'No'

wordMisspellingConstraint: 'No'

wordOcrConstraint: 'No'

snid: Linguamatics.FAMHIST

pt: Family History

of:

- alternative:

macroPt: Family History

macroSnid: Linguamatics.FAMHIST

of:

- phrase: [fam, hx]

- phrase: [family, history]

- father

- paternal

- grandmother

- mum

- dad

- famhx

- maternal

- grandad

- phrase: [fam, hist]

- uncle

- mom

- cousin

- phrase: [family, hist]

- sister

- phrase: [family, hx]

- brother

- grandfather

- aunt

- fhx

- mother

- word: {text: parent, morphoVariants: true}

- son

- daughter

- alternative:

quantifier: {minimum: 0, maximum: 0}

view: {layout: list}

of:

- word: {text: request, morphoVariants: true}

- word: {text: say, morphoVariants: true}

- word: {text: tell, morphoVariants: true}

- word: {text: inform, morphoVariants: true}

- word: {text: wish, morphoVariants: true}

- word: {text: want, morphoVariants: true}

- word: {text: demand, morphoVariants: true}

creator: minnamorati

/api;type=saved_query/problem_only/history_postmod.i2qy:

version: 5.3.1

creationDate: '2018-08-31 10:52:56'

query:

document:

- phrase:

allowOverlap: true

expression: |-

(function()

fam = i2e.get('Family', '')

i2e.set('Family', '')

return fam .. 'History'

end)()

maxWordGap: 2

ptSource: expression

showInHitColumn: false

unordered: true

of:

- alternative:

columnOptions: [Text]

label: Patient History

ptSource: label

showInColumn: false

showInHitColumn: false

of:

- alternative:

label: Year

ptSource: label

view: {layout: list}

of:

- word: {text: 19\d\d, matchType: Regexp}

- word: {text: 20\d\d, matchType: Regexp}

- alternative:

altFile: C:/Client/C$/customers/imo/Queries-v1.2/history_post_modified.txt

columnOptions: [Text]

of:

- phrase:

displayChildren: false

ptSource: label

of: [prior, to, admission]

- phrase:

displayChildren: false

ptSource: label

of:

- last

- alternative:

- time

- word: {text: year, morphoVariants: true}

- phrase:

displayChildren: false

ptSource: label

of:

- alternative: [until, during]

- word:

text: the

quantifier: {minimum: 0, maximum: 1}

- last

- phrase:

displayChildren: false

ptSource: label

of:

- alternative: [in, over, during]

- word:

text: the

quantifier: {minimum: 0, maximum: 1}

- past

- word: {text: earlier, ptSource: label}

- phrase:

displayChildren: false

ptSource: label

of:

- alternative:

quantifier: {minimum: 0, maximum: 1}

of: [and, but]

- alternative: [that, which]

- word:

text: it

quantifier: {minimum: 0, maximum: 1}

- alternative: [was, has]

- phrase:

displayChildren: false

ptSource: label

of:

- in

- alternative:

- school

- college

- university

- phrase:

displayChildren: false

of: [high, school]

- phrase:

displayChildren: false

ptSource: label

of:

- in

- alternative:

- childhood

- phrase:

displayChildren: false

of: [early, days]

- phrase:

displayChildren: false

ptSource: label

of:

- as

- a

- alternative: [child, teenager, youth, young]

- phrase:

displayChildren: false

ptSource: label

of: [at, the, age, of]

- phrase:

displayChildren: false

ptSource: label

of:

- at

- word:

text: a

quantifier: {minimum: 0, maximum: 1}

- young

- age

- phrase:

displayChildren: false

maxWordGap: 2

of:

- for

- word: {text: year, morphoVariants: true}

- alternative:

quantifier: {minimum: 0, maximum: 0}

view: {layout: list}

of:

- word: {text: today, morphoVariants: true}

- word: {text: yesterday, morphoVariants: true}

- word: {text: week, morphoVariants: true}

- word: {text: fortnight, morphoVariants: true}

creator: minnamorati
